# Divergent clinical outcomes after shared poison hemlock ingestion: evidence suggesting a dose-related effect

**DOI:** 10.1186/s12245-026-01159-4

**Published:** 2026-04-01

**Authors:** Asli Bahar Ucar

**Affiliations:** Department of Emergency Medicine, Marmara University Pendik Training and Research Hospital, Muhsin Yazicioglu mah no:10, Pendik, Istanbul, 34899 Türkiye

**Keywords:** *Conium maculatum*, Dose-dependent toxicity, Hemlock, Plant poisoning, Respiratory paralysis, Toxicology

## Abstract

**Background:**

Poison hemlock (*Conium maculatum*) ingestion is rare in contemporary emergency medicine but may result in rapidly progressive neuromuscular paralysis and respiratory failure. Because exposure is often unrecognized at presentation, emergency management decisions – particularly airway control – must frequently be made in the setting of diagnostic uncertainty.

**Case presentation:**

We report two patients who presented to the emergency department of Marmara University Pendik Training and Research Hospital in Istanbul, Türkiye after ingesting the same meal prepared from wild greens later identified as poison hemlock. Despite a shared exposure, their clinical courses differed markedly. One patient developed early respiratory muscle involvement with progressive ventilatory failure requiring endotracheal intubation and intensive care admission, whereas the second patient experienced only mild, self-limited neuromuscular symptoms managed with close observation.

**Conclusion:**

These cases illustrate how differences in ingested dose may lead to variable degrees of neurotoxicity following poison hemlock ingestion and emphasize that airway management decisions in the emergency department should be guided by physiological deterioration rather than diagnostic certainty.

## Background

**Fig. 1:**
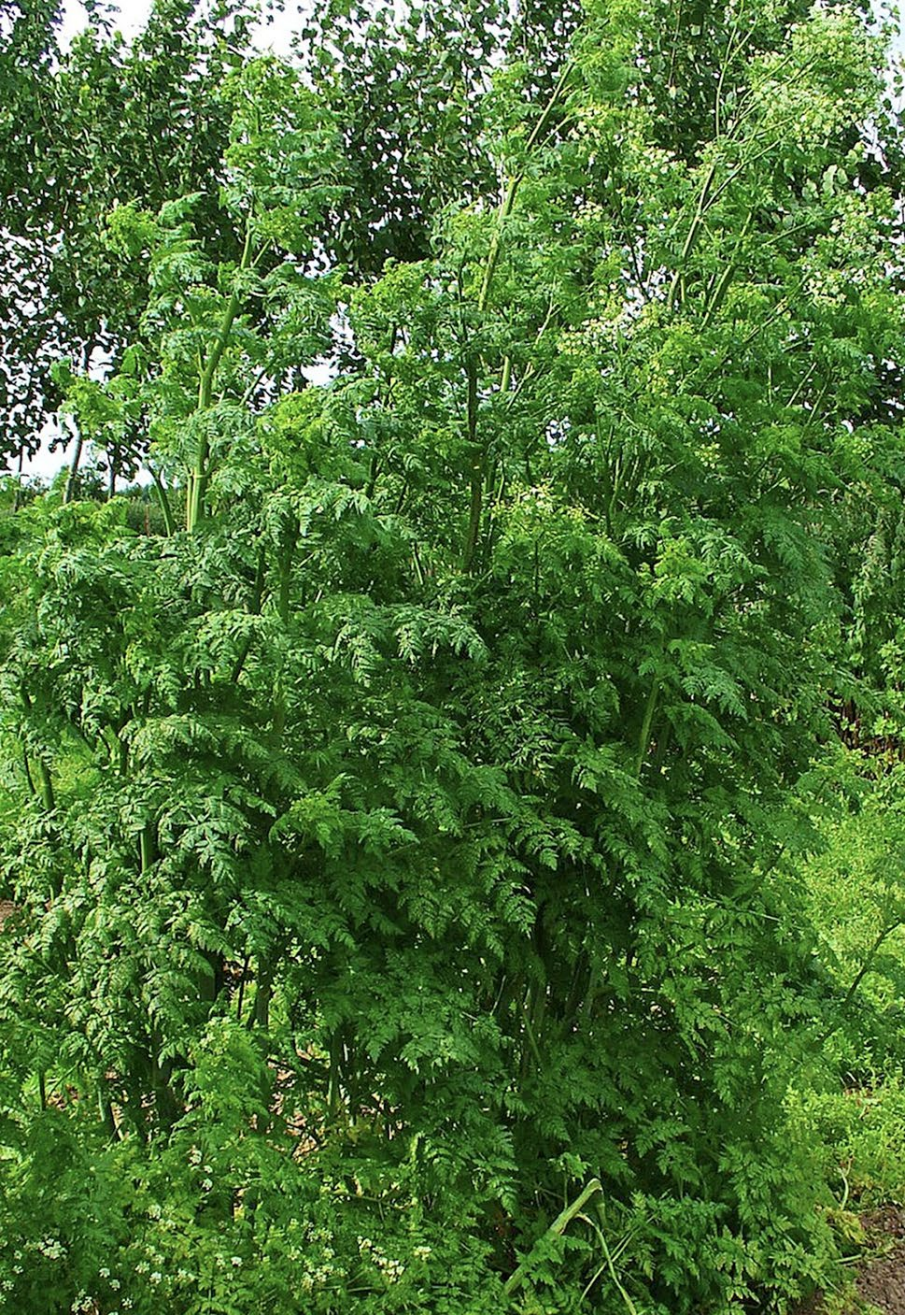
Conium maculatum (poison hemlock). Representative image demonstrating characteristic fern-like leaves and hollow stem. Image by H. Zell, licensed under CC BY-SA 3.0 [12]

Poison hemlock (*Conium maculatum*) is a highly toxic biennial plant containing piperidine alkaloids, primarily coniine and γ-coniceine, which act on nicotinic acetylcholine receptors and result in neuromuscular dysfunction [1, 2]. Although historically associated with the death of Socrates, poison hemlock is now infrequently encountered in modern clinical practice. When exposure occurs, it is typically accidental and related to misidentification of the plant as edible wild greens, particularly in rural or semi-rural settings [3]. The plant is characterized by a hollow stem that may display purple blotches and fern-like leaves (Fig. 1).

According to data from the Turkish National Poison Information Center, wild plant ingestion accounted for approximately 0.85% of all reported poisoning cases in Türkiye as of the most recent national report. Although relatively uncommon, such exposures remain clinically important due to their potential to cause severe neurotoxicity and respiratory failure, particularly when toxic plants are misidentified as edible species [4].

Clinical manifestations range from mild gastrointestinal or neurological symptoms to rapidly progressive paralysis and respiratory failure. Early symptoms may be nonspecific, and confirmatory toxicological testing is rarely available in the acute setting. As a result, emergency physicians must often rely on clinical trajectory and physiological parameters rather than etiologic certainty when making critical management decisions, including airway protection. Reports describing divergent clinical outcomes after a shared exposure are uncommon. We present two such cases to illustrate dose-dependent variability in neurotoxicity and its implications for emergency airway management.

## Case presentation

### Case 1

A 71-year-old man with a history of chronic obstructive pulmonary disease and hypertension was brought to the Emergency Department (ED) approximately 30 min after ingesting a meal prepared from wild greens. According to relatives, the dish was made from approximately 1 kg of leaves sautéed with oil and tomato paste. Within 10 min of ingestion, he developed rapidly progressive dyspnea, generalized weakness, and altered mental status.

Emergency medical services transported the patient on supplemental oxygen (8 L/min). On arrival to the ED, vital signs were notable for blood pressure (BP) 140/100 mmHg, heart rate (HR) 138 beats/min, respiratory rate (RR) 30 breaths/min, and peripheral capillary oxygen saturation (SpO_2_) 76% despite supplemental oxygen. His Glasgow Coma Scale score (GCS) was 8 (E2 V2 M4). Physical examination revealed marked hypersalivation and lacrimation with generalized weakness; no focal neurological deficits were identified, and pupillary light reflexes were preserved despite mild dilation.

Arterial blood gas analysis confirmed severe hypercapnic respiratory acidosis consistent with impending ventilatory failure (Table 1). This clinical pattern suggested evolving ventilatory failure rather than isolated central nervous system depression. Given the progressive respiratory compromise and concern for neuromuscular failure, endotracheal intubation was performed without waiting for definitive diagnostic confirmation. Computed tomography of the brain, chest, and abdomen revealed no acute pathology.

The patient was admitted to the intensive care unit (ICU) for mechanical ventilation and supportive care. He was successfully extubated after 40 h and discharged home without neurological sequelae.

### Case 2

The patient’s 68-year-old wife presented to the ED approximately 40 min after ingesting the same meal. She reported consuming only three spoonfuls and subsequently developed headache, dizziness, blurred vision, nausea, and mild limb weakness.

On evaluation, she was hemodynamically stable with BP: 125/85 mmHg, HR: 92 beats/min, RR: 20 breaths/min, and SpO_2_ 95% on room air. She was fully alert (GCS: 15). Neurological examination showed mild bilateral lower extremity weakness (4/5) with intact cranial nerve function. Laboratory evaluation did not reveal metabolic or respiratory abnormalities (Table 1).

Given the shared exposure and concern for possible delayed neuromuscular progression, she was admitted to the ICU for close observation. Her symptoms resolved over the following hours, and she was discharged after 24 h without complications.

Plant material brought to the ED by relatives was reviewed in consultation with the National Poison Center and identified as *Conium maculatum*.

No other individuals were reported to have consumed the same meal, and no additional related cases were identified.

**Table 1:** Clinical and laboratory findings

Parameter	Case 1 (Male)	Case 2 (Female)
Blood pressure (mmHg)	140/100	125/85
Heart rate (beats/min)	138	92
Respiratory rate (/min)	30	20
SpO_2_ (%)	76	95
Glasgow Coma Scale	8	15
pH	7.03	7.38
PaCO_2_ (mmHg)	82	39
HCO_3_⁻ (mmol/L)	20.7	23.4
Lactate (mmol/L)	5.9	Normal

Abbreviations: SpO_2_, peripheral capillary oxygen saturation; pH, arterial pH; PaCO_2_, arterial partial pressure of carbon dioxide; HCO_3_⁻, bicarbonate

## Discussion

Poison hemlock toxicity is generally considered uncommon in modern emergency medicine practice; however, national surveillance data indicate that wild plant ingestion accounted for approximately 0.85% of all reported poisoning cases in Türkiye according to the most recent Turkish National Poison Information Center report [4]. Although numerically low, such exposures remain clinically important due to the potential for rapid neuromuscular compromise and respiratory failure.

Beyond national data, *Conium maculatum* has a broad geographical distribution and is naturalized across Europe, North and South America, parts of Asia, Africa, Australia, and New Zealand [2]. Human intoxication cases have been reported internationally in both pediatric and adult populations [5, 6]. Published case reports and small case series from different regions describe a wide clinical spectrum, ranging from mild gastrointestinal and neurological symptoms to severe respiratory paralysis requiring mechanical ventilation [1, 5]. This variability has been attributed to differences in ingested quantity, plant maturity, alkaloid concentration, preparation methods, and individual susceptibility [2, 3].

Despite its global distribution, detailed human data correlating estimated ingestion amount with clinical severity remain limited. Most published reports describe clinical presentation without clearly documenting relative consumption quantities [5]. In this context, our cases suggest a probable dose-related difference in clinical severity following shared exposure to the same meal. While exact alkaloid concentrations could not be measured, the marked difference in ingested volume, combined with divergent respiratory outcomes, supports the toxicological principle that exposure dose is an important determinant of clinical effect [7].

Accidental ingestion may occur due to visual similarity between poison hemlock and edible wild plants such as *Chenopodium album*, particularly during seasonal harvesting. Variations in plant morphology across growth stages, environmental factors, and human error in identification may contribute to such incidents, even among individuals with prior foraging experience [2, 5].

Differences in clinical severity may also reflect individual patient factors. In the first case, advanced age and underlying chronic obstructive pulmonary disease may have reduced physiological reserve, potentially contributing to earlier respiratory decompensation.

Hemlock-induced neuromuscular failure should also be considered within the broader differential diagnosis of acute respiratory compromise in the emergency department. Organophosphate poisoning, for example, typically presents with prominent muscarinic findings such as bronchorrhea, bradycardia, and miosis, in addition to nicotinic manifestations [8]. In contrast, poison hemlock primarily acts through nicotinic receptor stimulation followed by neuromuscular blockade, often without the pronounced cholinergic secretory syndrome characteristic of organophosphate toxicity [2, 8]. Similarly, myasthenic crisis may present with progressive respiratory muscle weakness and hypercapnia; however, it is generally associated with a known history of autoimmune neuromuscular disease and lacks the acute toxic exposure history and autonomic features seen in plant alkaloid intoxication [9].

Other causes of acute neuromuscular respiratory failure, such as botulism [10] or severe metabolic disturbances, should also be considered in the appropriate clinical context; however, the abrupt onset following plant ingestion and the absence of characteristic features of these conditions supported a toxic alkaloid–mediated mechanism in our patients. Recognizing these distinctions is essential, as management strategies differ substantially, particularly regarding antidotal therapy and the timing of airway intervention.

The role of gastrointestinal decontamination in poison hemlock ingestion remains limited and context-dependent. Although the clinical benefit of activated charcoal has not been specifically established for *Conium maculatum*, current toxicology position statements recommend that single-dose activated charcoal may be considered when administered within the first hour after ingestion, provided the airway is protected and the risk of aspiration is low [11]. Its effectiveness declines with time as absorption progresses.

In the first case, rapid progression of neuromuscular weakness and progressive respiratory compromise raised significant concern for aspiration due to impaired protective airway reflexes. Given the evolving ventilatory failure and need for urgent intubation, activated charcoal was not administered prior to airway stabilization. Although administration after intubation may theoretically be possible, the interval from ingestion and the severity of neurotoxicity made a clinically meaningful benefit unlikely. In the second case, symptoms were mild and self-limited, without evidence of ongoing deterioration or significant systemic toxicity; therefore, the anticipated benefit of charcoal was considered minimal.

## Limitations

Confirmatory toxicological analysis was not available, and alkaloid concentrations could not be measured. Additionally, plant alkaloid content may vary considerably depending on plant maturity, environmental conditions, and preparation methods, potentially contributing to clinical heterogeneity. Nevertheless, diagnosis was supported by clinical presentation, identification of the ingested plant, and consultation with the National Poison Center. Despite these limitations, the cases provide valuable real-world evidence suggesting dose-related variability in human poison hemlock ingestion.

## Conclusion

Poison hemlock ingestion, although uncommon, can lead to rapidly progressive respiratory failure in the ED. These cases demonstrate how shared exposure may result in markedly different clinical outcomes depending on dose and individual susceptibility. Emergency physicians should prioritize early recognition of neurotoxic features and base airway management decisions on physiological deterioration rather than diagnostic certainty.

## Data Availability

The datasets generated and/or analyzed during the current study are available from the corresponding author on reasonable request.

## Abbreviations

BP: Blood Pressure
ED: Emergency Department
GCS: Glasgow Coma Scale
HCO_3_⁻: Bicarbonate
HR: Heart Rate
ICU: Intensive Care Unit
pH: Arterial pH
PaCO_2_: Arterial Partial Pressure of Carbon Dioxide
RR: Respiratory Rate
SpO_2_: Peripheral Capillary Oxygen Saturation
